# YouTube as a Patient Information Source for Gastrointestinal Reflux Disease

**DOI:** 10.7759/cureus.49118

**Published:** 2023-11-20

**Authors:** Veeramachaneni Naga Nyshita, Mahima Kuruvila, Swathi Galidevara, Akshay Sundaram, Shreya Sirohi, Mayank Singh

**Affiliations:** 1 Internal Medicine, Andhra Medical College, Visakhapatnam, IND; 2 Internal Medicine, Caribbean Medical University School of Medicine, Chicago, USA; 3 Internal Medicine, M. V. Jayaraman Medical College and Research Hospital, Hoskote, IND; 4 Internal Medicine, Kasturba Medical College, Manipal, IND; 5 Internal Medicine, Mahatma Gandhi Medical College and Research Institute, Aurangabad, IND; 6 Medicine and Surgery, Mahatma Gandhi Medical College and Research Institute, Aurangabad, IND; 7 Medical Services, Global Hospital, Mumbai, IND

**Keywords:** etiology, patient information source, youtube, gastroesophageal reflux disease, gerd

## Abstract

Introduction

Gastroesophageal reflux disease (GERD) affects a substantial portion of the global population, resulting in significant morbidity and impacting the quality of life. YouTube (YouTube, San Bruno, California) serves as a platform where medical professionals, individuals with personal experiences, and educational channels share their insights on GERD. However, with the vast amount of information available on YouTube, the question of credibility and reliability is a concern and, thus, is crucial to evaluate. This research paper aims to explore the impact of YouTube as a source of information on GERD. The aim of this study is to assess the quality and reliability of the information on YouTube about GERD.

Methodology

This cross-sectional observational study was conducted in June 2023. A questionnaire was designed using Google Forms (Google, Mountain View, California) with predetermined criteria such as characteristics of YouTube videos (time since uploaded, uploader, number of likes and comments); information about GERD (symptoms, investigations, treatment); and quality and reliability of information on YouTube about GERD using Global Quality Scale (GQS) and Reliability score. The Kruskal-Wallis Test was used to evaluate the difference in quality and reliability of information about GERD on YouTube based on the type of uploader.

Results

Out of 90 videos analyzed, 68 YouTube videos on GERD that met inclusion criteria were included in the study. The number of videos uploaded by hospitals was 28 (41.2%), those by doctors was 12 (17.6%), and the remaining by others (like pharmacists, patients, and non-medical personnel) was 28 (41.2%). A significant proportion of videos (88.24%) shared information pertaining to disease symptoms and cause/etiology. The videos uploaded by "others" had significantly higher (p<0.05) reach as assessed by the Video Power Index (VPI) compared to those uploaded by doctors and hospitals. However, there was no significant difference (>0.05) in the quality and reliability of videos uploaded by doctors, hospitals, and other sources.

Conclusion

Although the YouTube videos uploaded by doctors and hospitals had less reach among viewers compared to other uploaders (patients, news agencies, pharmaceutical companies, and others unrelated to healthcare), the quality and reliability of videos uploaded by doctors, hospitals, and other uploaders were of good quality and reliability and with no significant difference based on type of uploader. Healthcare organizations and government agencies should ensure that viewers have access to accurate and reliable information from social media like YouTube, which is crucial in their health decision-making.

## Introduction

Gastroesophageal reflux disease (GERD) affects a substantial portion of the global population, causing significant morbidity and impacting the quality of life [1]. The causes of GERD are multifactorial, including lifestyle choices, diet, obesity, pregnancy, and certain medical conditions contributing to its development [2]. Treatment options for GERD typically involve lifestyle modifications, such as avoiding trigger foods, losing weight if necessary, raising the head of the bed, and avoiding eating close to bedtime [3]. Medications, such as antacids, H2 blockers, and proton pump inhibitors, are also prescribed to manage symptoms through a reduction in stomach acid production.

YouTube (YouTube, San Bruno, California) serves as a platform where medical professionals, individuals with personal experiences, and educational channels share their insights in the context of GERD. Medical professionals should utilize YouTube to disseminate evidence-based information, educating viewers about the causes, symptoms, diagnosis, and treatment options for GERD. Patient testimonials and personal experiences on YouTube provide relatable perspectives, offering practical advice for managing GERD symptoms. Additionally, educational channels dedicated to health and wellness contribute to a comprehensive understanding of GERD through informative videos [4].

However, with the vast amount of information available on YouTube, it is crucial to critically evaluate the credibility and reliability of sources [5]. Misinformation, anecdotal advice, and unproven remedies can potentially mislead viewers and hinder the effective management of GERD. It is thus advised to consider the need for professional medical guidance and cross-referencing information from reputable sources [6].

The aim of this study is to assess the quality and reliability of the information on YouTube about GERD. The quality has been assessed using the Global quality scale(GQS) and the reliability has been evaluated using the Reliability score.

## Materials and methods

An observational study of the cross-sectional type was conducted on a single day, virtually, in June 2023. To maintain homogenous data, YouTube, a free online video-sharing platform largely used by the young adult population, was used to assess the information available about GERD. The top six keywords for GERD, namely "GERD", "GERD disease", "GERD causes", "GERD symptoms", "GERD treatment", and "GERD prevention" were taken into this study. The criteria for inclusion of YouTube videos in this study were: videos in the languages "English" or "Hindi", videos containing information about the disease GERD, videos of time duration ranging from one minute (less time duration to convey information) to twenty minutes (videos too long to hold the attention of viewers). Videos that did not meet the inclusion criteria and duplicate videos were excluded from the study. Each author was allotted one keyword, and they analyzed 15 top videos for the duration of ten days. A total of 90 videos were analyzed, and 68 videos that met the inclusion criteria were included in this study.

A questionnaire was made using Google Forms (Google, Mountain View, California) with several categories for assessment of these YouTube videos. These categories were: baseline characteristics of YouTube videos (time since uploaded, uploader, number of likes and comments); information about GERD (symptoms, cause, prevention, investigations, treatment, mortality, promotional content by pharmaceutical companies, patients sharing their own experience); and the quality and reliability of information on YouTube about GERD using Global Quality Scale (GQS) and Reliability Score. Videos that were factually correct as determined by the World Gastroenterology Organization (WGO) factsheet on GERD [7] were deemed true; otherwise, they were false. The quality and reliability of the YouTube videos on the aspects of GERD were assessed by Global Quality Scoring (GQS) and Reliability Scoring (RS)[8]. A pilot study of five videos was performed to test the questionnaire, changes for improvement were incorporated, and these videos were excluded from the study. 

Global Quality Score has five points and the most appropriate score is selected for a YouTube video: 1 - poor quality, poor flow of the site, most information missing, not at all useful for patients; 2 - generally poor quality and poor flow, some information listed but many important topics missing, of very limited use to patients; 3 - moderate quality, suboptimal flow, some important information is adequately discussed but others poorly discussed, somewhat useful for patients; 4 - good quality and generally good flow, most of the relevant information is listed, but some topics not covered, useful for patients; and 5 - excellent quality and excellent flow, very useful for patients [8].

The reliability score has five questions: Are the aims clear and achieved? Are reliable sources of information used? Is the information presented balanced and unbiased? Are additional sources of information listed for patient reference? Does it refer to areas of uncertainty? For each question, yes is scored as 1 and no as 0. The sum is calculated to give the final score [8].

The data was collected in Microsoft Excel (Microsoft, Redmond, Washington) and analyzed using SPSS software version 21.0 (IBM Inc., Armonk, New York).

## Results

A comprehensive evaluation was conducted on a total of 72 YouTube videos, wherein inclusion and exclusion criteria were applied, and the removal of repeated videos was done. A total of 68 refined videos were considered for analysis.

Table 1 elucidates the characteristics of analyzed YouTube videos. These videos collectively garnered 43,772,353 views, accompanied by 382,169 likes, 12,182 dislikes, and 27,722 comments. In terms of video upload timeline, it was observed that 14.7% of the videos were uploaded within a timeframe of 7 to 365 days preceding the study, while the remaining 85.3% had been available on the platform for over a year. Notably, the number of videos uploaded by hospitals was equivalent to those contributed by other sources and more prevalent than those uploaded by doctors. Other sources include sources other than doctors and hospitals, like patients sharing their own experiences, pharmaceutical companies, non-verified users, influencers, etc.

**Table 1:** Characteristics of YouTube videos analyzed

Time since uploaded	Number of videos (%)
More than a week to last one year (7-365 days)	10 (14.7%)
More than one year (>365 days)	58 (85.3%)
Popularity	Total number
Total no. of views	4,3772,353
Total no. of likes	382,169
Total no. of dislikes	12,182
Total no. of comments	27,722
Type of uploader	Number of videos (%)
Doctor	12 (17.6%)
Hospital	28 (41.2%)
Other	28 (41.2%)

Table 2 delineates the diseases covered within these videos. A significant proportion of videos (88.24%) shared information pertaining to disease symptoms and cause/etiology. The predominant topics covered included disease symptoms (88.24%), etiology (88.24%), complications (72.06%), treatment modalities (70.59%), preventive measures (58.82%), and description of surgical procedures (45.59%), in descending order of prevalence. Conversely, the provision of information regarding investigations/tests, mortality rates, rehabilitation, support groups, patient experiences, and recent advances was relatively limited.

**Table 2:** Information about GERD in the YouTube videos GERD - gastroesophageal reflux disease

Information	Number of videos (%)
Description of symptoms	60 (88.24%)
Information about cause/etiology	60 (88.24%)
Information about investigations/tests	23 (33.82%)
Information about prevention/vaccines	40 (58.82%)
Information about treatment	48 (70.59%)
Information about mortality	1 (1.47%)
Information about rehabilitation	1 (1.47%)
Information about support groups	1 (1.47%)
Information about people/patients sharing their experience	10 (14.71%)
Information about parents sharing experiences with their family members	4 (5.88%)
Description of complications	49 (72.06%)
Description of surgical procedures	31 (45.59%)
Description of recent advances in treatment	20 (29.41%)
Promotional content by pharmaceutical companies or by doctors	14 (20.59%)

Table 3 presents a comparison of the Global quality score (GQS), Reliability scores, and Video power index (VPI) based on the type of uploader. The VPI of other sources (videos uploaded by patients, non-doctors, and others) was significantly higher (p<0.05) than videos uploaded by doctors and hospitals. The median score for GQS was four for both doctors and hospitals, compared to three for other sources. There was no statistically significant difference (p>0.05) in the quality of videos uploaded by doctors, hospitals, or other sources. The reliability score for videos uploaded by doctors, hospitals, and other uploaders showed no difference (p>0.05). 

**Table 3:** Comparison of GQS, reliability score, and VPI based on type of uploader GQS - Global Quality Score; IQ1 - inter quartile 1 (lower quartile); IQ3 - inter quartile 3 (upper quartile); VPI - Video Power Index p-value <0.05 is significant

	Doctors (n=12)	Hospital (n=28)	Other (n=28)	Kruskal-Wallis test
	Median (IQ1, IQ3)	Median (IQ1, IQ3)	Median (IQ1, IQ3)	
VPI	99.36 (9.89, 270.27)	29.9 (8.05, 94.88)	110.29 (19.15, 730.02)	p-value = 0.047
GQS	4 (3.25, 5)	4 (3, 5)	3 (3, 4.75)	p-value = 0.355
Reliability Score	4 (4, 5)	4 (3, 5)	4 (3, 4)	p-value = 0.199

## Discussion

Gastroesophageal reflux disease (GERD) is a prevalent complex condition that is chronic and sometimes progressive [9]. GERD presents with a range of symptoms and complications while requiring the initiative of self-management. With adequate lifestyle changes and an understanding of how to use educational resources thoroughly, patients have the ability to combat this disease with knowledge. Various Studies have successfully proved and cited the benefits of resources such as YouTube as an asset when exploring a particular disease [10,11]. A patient is able to explore the possible cause and etiology, clinical presentation, symptoms, diagnosing tools and tests, prevention and vaccines, treatment options, complications, and support and management options. [10]

Our study had several important findings. The analysis revealed that within the majority of the videos analyzed, 58 (82.9%) of them were uploaded more than a year ago; this suggests that perhaps the YouTube content available for gastroesophageal reflux disease is outdated. These findings are similar to the findings of a similar study conducted by Holge et al., which reported 40 videos (78.44%) [11]. Outdated information can be misleading due to advances in care and support with further research. This can prove to be harmful for a patient who is seeking guidance online while attempting to make a well-informed decision.

As mentioned previously, the quality and reliability of the YouTube videos on the aspects of GERD were assessed by Global Quality Scoring (GQS) and Reliability Scoring (RS); we also observed Video Power Index (VPI) among the various sources to critically analyze the quality and reliability of the Youtube Videos. A previous study conducted by Osman et al. reported that there is an overall lack of quality among YouTube videos when used as a healthcare resource for patients [12]. Our study reported a GQS and Reliability Score score of 4. These scores represent a consistently higher quality of videos attributed by doctors, hospitals, and other sources among the videos analyzed in our study. This was a similar finding in a study conducted by Caroline et al., which found that physicians and healthcare organizations also produced the highest quality of videos [13]. This is beneficial for the patients who are researching YouTube for videos focused on GERD as they prove to be of higher quality and reliability.

The most concerning discrepancies were reflected while examining the Video power index (VPI), with scores of 99.36, 29.9, and 110.29 for doctors, hospitals, and other sources, respectively. The resultant p-value of 0.047 substantiates a statistically significant difference in Video power index (VPI) among the various sources. This was also seen in a study conducted by Mylavarapu et al., which also reflected scores of 84.01, 12.595, and 184.71 for doctors, hospitals, and other sources, respectively, with a p-value of 0.038 [14].

A majority of the analyzed videos in our study cover the symptoms and treatment of GERD. (88.24% and 70.59% of videos, respectively). A majority of the analyzed videos in our study cover the symptoms and treatment of GERD. (88.24% and 70.59% of videos, respectively). This information is comparative to a similar study that was performed by Lee et al., which analyzed videos having a greater reach with views of over 35,220,141 [15]. A large percentage of 70.59% of the videos in this study provided information regarding treatment, while a study by Kaya depicted only 21% [16].

Upon statistical analysis of data, it was found that the quality of videos posted by doctors and hospitals is nearly the same. There is no difference between these groups, and this was clear in the 28 (41.2%) videos analyzed each. The Reliability Score of each of these groups also stands equal. Considering that there is no difference in scores between the groups, it can be interpreted as YouTube becoming more popular and the increased ease with which laypeople can easily access and produce content.

Limitations

This study had several limitations. First, The videos that were not in English or Hindi were excluded. This can result in the exclusion of some videos in the local language. Second, each author evaluated 15 videos, owing to the fact that an average person, while browsing, would generally not look beyond 15 posts as it tends to get overwhelming. Third, the calculation of the Global Quality Score and Reliability Score may vary from person to person. Fourth, the results of the search terms can change (adding new and removing old videos), making the YouTube platform very dynamic. Finally, the sample size was limited, but it is rare for any viewer to go through 80+ videos on YouTube.

## Conclusions

The YouTube videos uploaded by doctors and hospitals had less reach among viewers compared to other uploaders (patients, news agencies, pharmaceutical companies, and others unrelated to healthcare). The quality and reliability of videos uploaded by doctors, hospitals, and other uploaders were of good quality and reliability and with no significant difference based on the type of uploader. Healthcare organizations and government agencies should ensure that viewers have access to accurate and reliable information from social media like YouTube, which is crucial in their health decision-making.

## References

[1] El-Serag HB, Sweet S, Winchester CC, Dent J (2014). Update on the epidemiology of gastro-oesophageal reflux disease: a systematic review. Gut.

[2] Fox M, Gyawali CP (2023). Dietary factors involved in GERD management. Best Pract Res Clin Gastroenterol.

[3] Austin GL, Thiny MT, Westman EC, Yancy WS Jr, Shaheen NJ (2006). A very low-carbohydrate diet improves gastroesophageal reflux and its symptoms. Dig Dis Sci.

[4] Ventola CL (2014). Social media and health care professionals: benefits, risks, and best practices. P T.

[5] Aydin MF, Aydin MA (2020). Quality and reliability of information available on YouTube and Google pertaining gastroesophageal reflux disease. Int J Med Inform.

[6] Sinnenberg L, Buttenheim AM, Padrez K, Mancheno C, Ungar L, Merchant RM (2017). Twitter as a tool for health research: a systematic review. Am J Public Health.

[7] Hunt R, Armstrong D, Katelaris P (2017). World Gastroenterology Organisation global guidelines: GERD global perspective on gastroesophageal reflux disease. J Clin Gastroenterol.

[8] Ozturk T, Gumus H (2022). YouTube as an information and education source for early orthodontic treatment. Am J Orthod Dentofacial Orthop.

[9] Velagala NR, Velagala VR, Lamture Y (2022). The spectrum of treatment modalities for gastroesophageal reflux disease (GERD): a narrative review. Cureus.

[10] Zhang X, Yang Y, Shen YW (2022). Quality of online video resources concerning patient education for neck pain: A YouTube-based quality-control study. Front Public Health.

[11] Holge S, Gogikar A, Sultana R, Rathod U, Chetarajupalli C, Laxmi Supriya Y (2023). Quality and reliability of YouTube videos on myocardial infarction: a cross-sectional study. Cureus.

[12] Osman W, Mohamed F, Elhassan M, Shoufan A (2022). Is YouTube a reliable source of health-related information? a systematic review. BMC Med Educ.

[13] Diers CS, Remvig C, Meteran H, Thomsen SF, Sigsgaard T, Høj S, Meteran H (2023). The usefulness of YouTube videos as a source of information in asthma. J Asthma.

[14] Mylavarapu M, Maheta D, Clarke S, Parmar K, Mohammed M, Vuyyuru CS (2023). Diabetes mellitus on YouTube: a cross-sectional observational study to assess the quality and reliability of videos. Cureus.

[15] Lee KN, Tak HJ, Park SY, Park ST, Park SH (2022). YouTube as a source of information and education on endometriosis. Medicine (Baltimore).

[16] Kaya C, Usta T, Baghaki HS, Oral E (2021). Relation between educational reliability and viewer interest in YouTube® videos depicting endometrioma cystectomy surgical techniques. J Gynecol Obstet Hum Reprod.

